# Optimization and Validation of a New Microbial Inhibition Test for the Detection of Antimicrobial Residues in Living Animals Intended for Human Consumption

**DOI:** 10.3390/foods10081897

**Published:** 2021-08-16

**Authors:** María Jesús Serrano, Luis Mata, Diego García-Gonzalo, Alejandra Antón, Pedro Razquin, Santiago Condón, Rafael Pagán

**Affiliations:** 1 Instituto Agroalimentario de Aragón-IA2, Universidad de Zaragoza-CITA, 50013 Zaragoza, Spain; mjserran@unizar.es (M.J.S.); dgarcia@unizar.es (D.G.-G.); scondon@unizar.es (S.C.); 2 Department of R&D, ZEULAB S.L., 50197 Zaragoza, Spain; lmata@zeulab.com (L.M.); aanton@zeulab.com (A.A.); prazquin@zeulab.com (P.R.)

**Keywords:** antibiotics, sulfonamides, antimicrobials, blood, in vivo, biological test

## Abstract

Even though antibiotics are necessary in livestock production, they can be harmful not only due to their toxicity, but also in view of their contribution to the emergence of antimicrobial resistance. Screening tests based on microbial growth inhibition appeared to be useful tools to prevent its entry into the food chain. They have nevertheless been traditionally carried out post mortem, leading to great economical loss and harm to the environment in case a positive sample is found. Hence, the objective was to evaluate the use of a screening test as an ante mortem alternative for the detection of antibiotic residues in meat: thus, Explorer^®^-Blood test was optimized and validated. After adapting the procedure for matrix preparation, the assay parameters were assessed from 344 antibiotic-free blood serum samples. Limits of Detection (LoDs) were defined by spiking blood serum with several of the most common antimicrobials used in veterinary practice. LoDs were similar to those obtained for meat and were at or below the maximum residue limits set by EU legislation for muscle. Analyses of in vivo injected samples, previously characterized by LC-MS/MS, demonstrated the method’s accuracy and proved that Explorer^®^-Blood can be considered a suitable alternative to conventional post mortem screening methods.

## 1. Introduction

Antimicrobial detection tests are conventional screening tools used in slaughterhouses to prevent the entry of antimicrobial residues into the food chain. The occasional appearance of antibiotic or bacteriostatic residues is a problem of major worldwide concern, as such residues can lead not only to toxicity for humans, but also to the emergence of antimicrobial resistance (AMR) [1,2]. In particular, antibacterial residues that contaminate meat can cause allergic reactions, can lead to dysbiosis of the gastrointestinal flora and can enhance dissemination of AMR, not only in the environment but also inside the gut, leading to antibacterially resistant communities in our intestinal flora [3,4,5,6]. Even though the development of AMR is the most critical issue as it is responsible for the loss of effectiveness of antimicrobials against common infectious diseases, less studied aspects, such as the unknown toxicity of their derivates after cooking meat [7] or the presence of residues of oxidative antimicrobials that disrupt the endogenous antioxidant system [8], should likewise be taken into consideration.

In order to address this problem and create a safe environment for consumers, the European Union (EU) has established an extensive legislative framework by restricting the authorization of medicinal products allowed for veterinary use [9], by determining maximum residue limits (MRLs) [10], by setting safe withdrawal periods [11], by monitoring plans [12] and by establishing a series of analytical methods for the testing of official samples [13].

A wide range of methods for the analysis of antibiotic residues in meat are currently available. In Europe, methods for official control are classified as screening and confirmatory methods. The most common surveillance programs for antibiotic residue control start by screening a large number of samples in a short time with easy, inexpensive methods [14]. Screening methods must detect a broad spectrum of antimicrobials at the regulatory levels; ideally, no more than 5% false compliant results should be accepted. Moreover, presumptive non-compliant results must be confirmed with a suitable validated method [13].

Among recommended screening methods, several commercialized tests for the detection of antibiotics in food matrices based on growth inhibition of microorganisms are available. Many of them inhibit the growth of microorganisms such as *Geobacillus stearothermophilus* when antibacterial residues are present in a sample [14]. Further simplification and automatization of this kind of assays has been proposed in recent years. An example of such methods is the Explorer^®^ test, initially validated for the analysis of muscle [15] and eggs [16]. Explorer^®^ tests are presented in a tube in which a specific detection media is spread with the target microorganism. A growth indicator is additionally included in the medium, usually based on change of pH or redox potential. When the target microorganism grows (thereby indicating that the sample contains no antibiotics, or that their residues lie below the method’s limit of detection), the test medium colour changes from blue/purple to yellow/orange. However, if the sample contains antimicrobials, metabolism of the bacteria is stopped or slowed down and no colour change or only a partial colour change is visible.

Surveillance of antibiotic residues in foods of animal origin is carried out post mortem. If a positive sample is found to have amounts of antibacterial residues that are over the limits set by legislation (MRLs), carcasses must be confiscated and destroyed. Such an event has devastating repercussions for the farmer, who has to face substantial financial loss caused by severe fines, as well as the investment lost in breeding animals of no value. In addition, one must consider the harmful environmental impact of breeding livestock that ends up as waste, associated with the misuse of input resources and the release of contaminating output such as gas emissions, manure/slurry, residual water and the challenge of destroying the carcasses [17].

**Figure 1:**
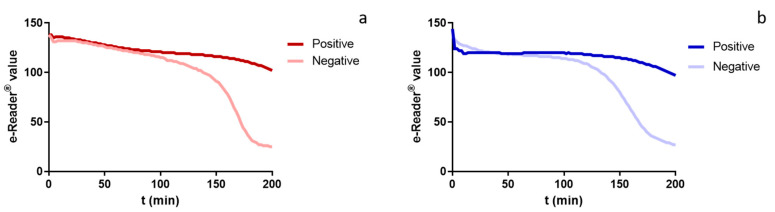
Colour change kinetics registered by e-Reader^®^ for positive (dark line) and negative (light line) blood serum samples analysed with Explorer^®^-Blood test (**a**) and muscle samples analysed with Explorer^®^ (**b**).

The analysis of antimicrobial residues in animals prior to slaughter has thus been attracting increased attention in recent years [18,19]. Several issues need to be solved, however, before implementing a methodology for in vivo testing for antibiotics. First of all, the selection of the most suitable matrix (tissue or biological fluid) is an essential requirement. A matrix for antibiotic detection should be easy to collect and should be representative of the level of antimicrobial substances found in edible tissues (e.g., muscle). To ascertain this, several studies comparing the concentration of antibacterial compounds in body tissues and fluids have been published [20,21,22,23,24]. A most recent study carried out on pigs demonstrated that blood serum was the most suitable matrix for laying the bases of a new in vivo antimicrobial detection test [25], as the concentration of antimicrobial molecules in blood serum showed an acceptable equivalence with that found in muscle (Figure 1b). Moreover, the collection of blood from living animals is a simple practice that is commonly carried out in farms. 

The implementation of systems for ante mortem screening of antibiotics in livestock by testing blood serum samples could help to overcome the aforementioned limitations of post-mortem analysis. In order to be put into practice as a routine plan, the method needs to be rapid and affordable, allowing for decision-making within a few hours. However, no simple and automatic method has yet been adapted for the screening of antimicrobials in blood. 

Therefore, the aim of this study was to optimize and validate Explorer^®^-Blood, based on the utilization of the Explorer^®^ test, as an ante mortem method for the detection of antimicrobial residues in blood samples obtained from living animals, in order to prevent their presence in carcasses and meat prior to their commercialization.

## 2. Materials and Methods

### 2.1. Blood Serum Samples

#### 2.1.1. Antibiotic-Free Blood Serum Samples

A total of 344 blank blood samples coming from antibiotic-free farms of Zaragoza province (Spain) were collected for the purpose of establishing the new method’s reading parameters (cut-off and reading time) and false positive rate.

#### 2.1.2. Antimicrobials Choice for Validation

**Figure 2:**
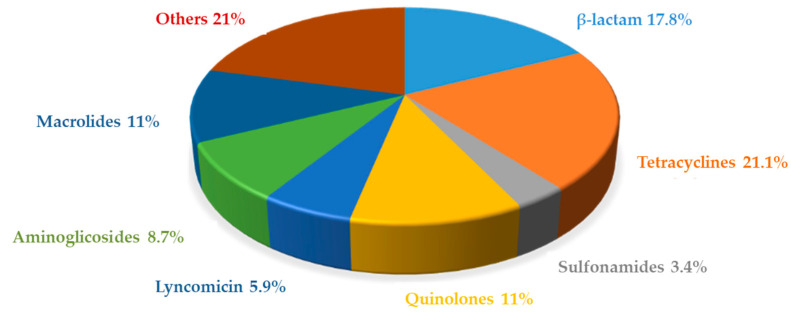
Distribution of authorized antimicrobial drugs for use in pig farming in Spain (CIMAVET, 2020).

Microbial inhibition tests are regarded as multi-class methods, that means they can detect a broad spectrum of antimicrobial groups. The Explorer^®^ test is capable of detecting a wide range of over 50 antimicrobial substances. A complete validation, including all the antimicrobials featuring an EU-MRL in muscle, would be unaffordable in terms of time and expense [26]. In order to select a reasonable number of molecules to be included in the validation, only representative molecules were taken into account. The criterion used for that purpose was to select one or two molecules from each of the main antimicrobial families. Molecules from each antimicrobial family were selected according to their potential frequency of use in livestock, specifically in pig farming. Thus, the antimicrobial drugs authorized for use in pig farming in Spain were obtained from CIMAVET database [27]. The frequency distribution of the antimicrobial families included in the authorized drugs list is shown in Figure 2. Tetracyclines, β-lactams and quinolones were present in half of the authorized drugs. 

**Figure 3:**
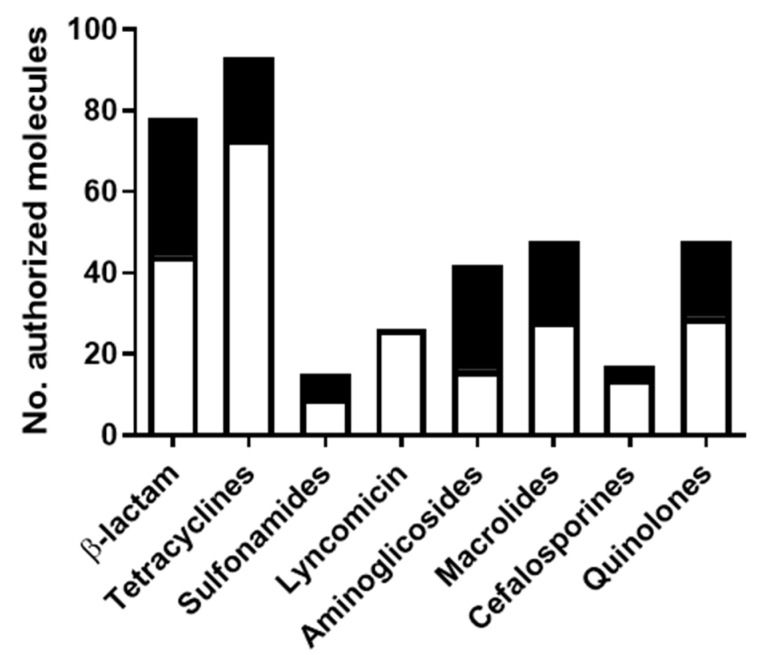
Number of approved products from the main authorized families for veterinary practice in pig farming [27]. Black bars show the total number of approved molecules of the main antimicrobial families, and white bars represent the number of those most frequently authorized: amoxicillin for ß-lactam; oxytetracycline and doxycycline for tetracyclines; sulfadiazine for sulfonamides; lyncomicin as the only molecule included in its family; neomycin and apramycin for aminoglicosides; tylosin for macrolides; ceftiofur for cefalosporines and enrofloxacin for quinolones.

Moreover, this database contains the most frequently approved molecules from each family that were selected as representative molecules for our validation study. Figure 3 shows the molecules most frequently authorized within each of the major families previously cited. As an exception, phenicols and quinolones were not included in the study because of their low inhibitory ability towards *G. stearothermophilus* [14].

#### 2.1.3. Antimicrobial Standards and Spiked Blood Serum Samples

Antibiotics (amoxicillin, cephalexin, ceftiofur, oxytetracycline, doxycycline, neomycin, apramycin, tylosin and lincomycin) and sulfonamides (sulfamethazine and sulfadiazine) used as standards were supplied by Sigma-Aldrich (Poole, UK). For each of them, a stock solution of 1 mg mL^−1^ was made in water or methanol (depending on solubility specifications given by the supplier), and aliquots were kept at −20 °C for no more than 2 months.

Spiked blood serum samples were used for the establishment of the limits of detection (LoD). Working dilutions were freshly made on a daily basis. Standards were prepared by spiking blank blood sera with the appropriate dilution to obtain the indicated levels for each molecule (Table 1), using as a reference the EU-MRLs and the LoDs described for muscle when tested with the Explorer^®^ test [15]. No more than 10% of antimicrobial dilution was added to the blood serum sample in order to avoid significant changes in the matrix composition.

#### 2.1.4. Blood Samples with Antibiotics Injected In Vivo

Blood samples with antibiotics injected in vivo were obtained from the sample bank built by Serrano et al. [25] from antibiotic-treated and untreated pigs. All the blood serum samples were analysed by LC-MS/MS to determine the concentration of antibiotic residues using a Waters Acquity Liquid Chromatograph (Waters Corporation, Milford, MA, USA). These blood samples were used for the validation of the test once the LoDs were determined with spiked samples.

### 2.2. Sample Preparation

As previously described, swine blood samples were obtained from the sample bank and local farms. Whole blood, blood diluted in buffer and serum were initially tested with Explorer^®^ tubes to evaluate their suitability for antimicrobial analysis. To obtain serum, after collection, blood was coagulated at room temperature for 1 h, following the protocol established by Serrano et al. [25]. The coagulum was then removed, and serum was centrifuged at 3000 rcf for 10 min on a Heraeus Megafuge 1.0 R centrifuge (Heraeus, Hanau, Germany). Blood sera were stored at −20 °C until analysis. 

In addition, several conditions for sample preparation were tested, bearing in mind that the sample preparation process should be kept as simple as possible in order to be performed at the farms or slaughterhouses where time and laboratory equipment are not available. Blood dilution in PBS, serum obtention and additional thermal treatments to deproteinize serum were evaluated by adding 100 µL of sample directly to the Explorer^®^ test tube.

### 2.3. Explorer^®^-Blood Test

#### 2.3.1. Description

Explorer^®^ (Zeulab, S.L., Zaragoza, Spain), is a qualitative test supplied in an ampoule format, based on the inhibition of microbial growth of *G. stearothermophilus*. Each tube test contains a nutrient medium spread with the target bacteria and a pH indicator. When the test is incubated at 65 °C, spores germinate and cells grow, thereby producing acid and changing the medium colour from blue to yellowish. Colour changes are monitored by an e-Reader^®^ device (Zeulab, S.L., Zaragoza, Spain) as an indication of microorganism metabolism. When the sample contains antimicrobials at levels higher than the LoD delays in the acidification kinetics, no colour changes are observed and consequently, samples are interpreted as positive.

#### 2.3.2. Procedure

100 µL of sample were added to an Explorer^®^ test tube with a micropipette, and then pre-incubated at room temperature for 20 min to allow sample diffusion through the medium. Sample was subsequently discarded, and the tubes were washed once with distilled water. Water excess was removed by turning the tubes upside down over adsorbent paper. Finally, the tubes were incubated in the e-Reader^®^ device at 65 °C. Results were obtained after approximately 180 min.

### 2.4. Parameter Setting and Validation of the Explorer^®^-Blood Test

#### 2.4.1. Cut-Off Level

To determine the optimal moment to halt the assay, kinetics of colour changes were monitored for negative and positive samples along incubation time with the e-Reader^®^ device. To establish the cut-off level of the test, 344 blood serum samples from pigs grown in antibiotic-free farming conditions were analysed, and Equation (1) was applied at different incubation times.

Equation (1). Cut-off level of the Explorer^®^-Blood test.

(1) $$\mathrm{Cut-off}=\bar{x}\pm 3SD$$

Samples displaying a result above the determined cut-off were considered positive.

#### 2.4.2. Limits of Detection (LoD)

For practical reasons, LoDs were determined by spiking blood sera with chosen antimicrobial substances at different levels. For the preparation of the spiked samples, at least 2 different standard solutions were used for each substance. To calculate the LoD, each antimicrobial was analysed at 3–4 levels around the EU-MRL [10] for muscle, and at a level matching the LoD expected for that compound. When the test did not detect an expected concentration as positive, a higher level was added to the study. Table 1 summarizes the substances and levels tested in the study.

Since there are no specific guidelines for the validation of screening methods for the detection of antibiotics in blood serum, a specific guideline for the validation and LoD establishment of microbial inhibition tests for the detection of antibiotics in milk (ISO 13969: 2003) [28] was adapted. It indicates that when interpretation is performed with an objective reading system, only 3–5 replicates are required for each substance/level combination. 

Therefore, at least 3 replicates per concentration level (Supplementary Materials, Table S1) were tested. The LoD was considered as the lowest level in which all repetitions gave a positive result. However, to obtain a more reliable estimation, up to 8 replicates were performed at the LoD level.

#### 2.4.3. False Positive Rate

False positive rate was determined by testing 344 blood serum samples from pigs grown in antibiotic-free farming conditions. Results above the established cut-off would indicate a false-positive result and require additional confirmation.

#### 2.4.4. Validation with Blood Samples Containing Antibiotics Injected In Vivo

Blood samples with different in-vivo-injected levels of oxytetracycline, sulfamethoxypyridazine and amoxicillin were analysed with Explorer^®^ test, and results were compared to those obtained by LC-MS/MS as described by Serrano et al. [25]. 

### 2.5. Statistical Analysis

Results were obtained from at least 3 replicates and are presented as the mean ± standard deviation. The PRISM^®^ program was used for data processing and representation, as well as for statistical analysis (GraphPad Software, Inc., San Diego, CA, USA). 

## 3. Results and Discussion

### 3.1. Matrix Preparation and Test Procedure

According to the results obtained by Serrano et al. [25], an acceptable equivalence exists between the concentration of antibiotics in blood and in muscle for all the antibacterial compounds studied (oxytetracycline, sulfamethoxypyridazine, enrofloxacin and amoxicillin), at a range of concentrations between the LoD and more than 4 (oxytetracycline) and 40 (amoxicillin and sulfamethoxypyridaxine) times the EU-MRL. Hence, blood was selected as the most suitable matrix for in vivo antimicrobial detection. Several commercial tests are available on the market for the screening of antimicrobials in edible products, but until now only the Explorer^®^ test has been optimized to be performed with an automatic system for reading and interpretation of results [15,16]. Therefore, due to its ease of use and automatic interpretation, the Explorer^®^ test was chosen to be adapted for the detection of antimicrobials in blood. Its features make it suitable for use in farms and slaughterhouses, where the time to obtain results is limited and laboratories are not readily available.

In a first step, blood was directly added to Explorer^®^ tubes in order to evaluate its behaviour. However, as this test is based on the colour change of the medium, blood tonality modified the appearance of the test to the point of hindering interpretation of results; and an additional step sample preparation was therefore required to ensure the test’s accurate performance. Thus, several conditions for sample preparation were tested: blood serum collection, blood dilution in buffers and additional thermal treatments to deproteinize serum (data not shown) were evaluated. All the preparation methodologies showed a similar improvement in acidification kinetics compared to the direct addition of blood. Nevertheless, although blood dilution in buffer showed similar kinetics, a loss in the test’s detection capability due to the dilution effect could be expected. Moreover, blood serum is easy to obtain compared to methodologies for deproteneization, and the acidification kinetics showed curve shapes (Figure 1a) similar to those obtained with meat juice (Figure 1b), one of the usual matrices for which the Explorer^®^ test and the e-Reader^®^ device were previously optimized. Thus, blood serum extraction was selected as the more suitable sample preparation method, since it consequentially reduced time and workload for the new Explorer^®^-Blood test.

### 3.2. Parameter Settings

Microbial inhibition tube tests are qualitative screening methods, which means that they give binary results (positive or negative). Even though these kinds of tests are ready to use and only require an incubator, they need to be halted at an adequate point in time to avoid over- or under-incubation and, thus, to obtain best results in terms of sensitivity. Moreover, a visual reading of results can lead to misinterpretation, as well as discrepancies between lab results and those obtained by users [29]. To overcome these issues, Mata et al. [15] proposed the automation of assays by coupling the microbial test (Explorer^®^) with an automatic device (e-Reader^®^) designed to detect antimicrobials in muscle. Automation was later extended to the detection of antimicrobials in milk [30,31] and eggs [16]. This device includes a thermostatic incubator adapted to the test tubes, and an optical system to monitor colour change. Both are integrated with a software that controls device functions and automatically detects the end-time of the assay to provide an objective result. Handling thereby becomes so easy that analysis can be performed at any location (on a farm, or during lairage time at the slaughterhouse), even by untrained staff. 

When an objective reading value is obtained through a reader device, it is necessary to establish a cut-off level as a binary criterion for the interpretation of results. In addition, setting the assay end-time is crucial in order to obtain best performance. To establish the method’s assay end-time and cut-off, blank blood serum samples were assayed with the Explorer^®^-Blood test and colour change kinetics were monitored along the incubation time with e-Reader^®^. Figure 1a shows the kinetics of a blank blood serum sample (free of antimicrobials) and the kinetics of a sample containing antimicrobials at concentrations lying over the test’s LoD. Kinetics described for the blank sample showed a typical sigmoid curve corresponding to the acidification of the medium as a consequence of the target microorganism growth when antimicrobials are not present in the sample. The highest e-Reader^®^ values (around 140 units) were obtained at the beginning of the assay, when colour was blue-purple, and the lowest (around 25 units) when colour shifted to yellow. Samples containing antibiotics at concentrations lying over the LoD were not able to reach a complete acidification during the assay; therefore, the kinetics could not reach the lowest values (or they were reached after a considerable delay). 

#### 3.2.1. End-Time

The end-time of the assay was established after having studied the kinetics of 8 blank blood sera: it is the moment when the test must be stopped and results can be read. After having evaluated the curves, it was decided to conclude the assay when e-Reader^®^ results reached a value of 35, corresponding with the value at which the kinetics start to slow down the slope. It is the moment when the optimal relationship between the assay’s highest sensitivity and the lowest variability is achieved. If the test is halted too soon (i.e., under-incubation), a higher sensitivity can be obtained, but the assay’s overall variability increases. To exemplify this increase in variability, 8 blank serum samples were analysed with Explorer^®^-Blood: the standard deviation (SD) obtained for e-Reader^®^ values was 5.0 when the assay was halted at a mean value of 35. However, SD increased up to 8.8 when the assay was concluded at a mean value of 50, indicating an increase in variability associated with a shorter assay time. Consequently, if the assay was under-incubated, the need arose to set a higher cut-off value in order to reliably obtain positive results while compensating for the increase in readout variability and reducing the false-positive rate [19]. Conversely, with longer incubation times, variability was reduced (SD = 1.7 when the assay was halted at a mean value of 25). Consequently, the test’s sensitivity was also reduced, particularly in the case of bacteriostatic molecules, thereby increasing the risk of obtaining false-negative results. This shows that detection capability decreases when the incubation time is too long. However, as the endpoint of the test is based on the optical measure of the negative control and does not depend on a previously defined time extent, this issue does not affect the assay’s precision [29]. 

#### 3.2.2. Cut-Off Level

**Figure 4:**
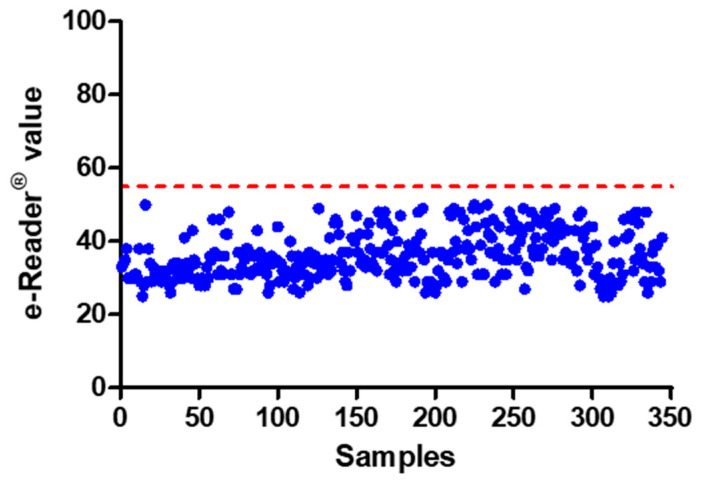
e-Reader^®^ values obtained for the 344 blood serum samples from animals grown in antibiotic-free farming conditions. The dashed line shows the cut-off value.

Once the assay end-time was established, it was necessary to calculate the point in time at which samples are considered positive: the cut-off level. The cut-off of a screening test is the response or signal which indicates that a sample contains an analyte at or above the target concentration, thereby ensuring discrimination between positive and negative samples. In order to establish the cut-off of the assay, a total number of 344 serum samples were analysed. The e-Reader^®^ values for each one of those samples at the point in time when the negative control reached the assay end-time are represented in Figure 4. An average value of 36 was obtained, and SD was around 6. By applying a safety factor of 3 times the SD (for a 99.7% level of confidence) and rounding up, the cut-off value was established at 55, signifying that all samples with colour values over 55 are considered positive. 

The cut-off depends on the method’s desired safety level. When the aim is to avoid assigning any non-compliant sample as negative, the established cut-off has to be reliable, but as low as possible. This criterion is proposed by the CRL (Community Reference Laboratories Residues) [32] guidelines for the validation of screening methods to be used in official controls (safety factor = 1.64 times the standard deviation from the average value of negative samples). In this case, the method’s sensitivity increases, but the false-positive rate can also rise, which means that true negative samples could be assigned as positive. Such a situation would require further confirmatory analyses, and the total cost and time of analysis would consequently increase. On the other hand, when a low number of false non-compliant results is required, for instance due to difficulties in the implementation of a confirmatory method, the cut-off level can be set higher (to obtain a higher level of confidence when assigning positive results). In this case the false-positive rate would decrease, but detection capability might be compromised due to the detection of certain antibiotics. Hence, it is important to find the right balance according to one’s intended purpose. In the present study, it has been selected a high level of confidence (safety factor = 3 times the standard deviation from the average value of negative samples), since this test is destined to be used at farm level or in stages prior to slaughter where reliable confirmatory methods are not available. 

#### 3.2.3. False Positive Rate

After the establishment of the test’s end-time and cut-off level, the results obtained after analysing the 344 blank blood sera were studied. None of the blank samples yielded a positive result (Figure 4). Therefore, a 0% false positive rate was described for the new method.

### 3.3. Limits of Detection (LoDs) in Swine Blood Serum

Once the testing procedure and the cut-off criteria are established, one must assess whether the test’s limits of detection are fit-for-purpose. Ideally, for in vivo screening, LoDs should be equal to or lower than those obtained in muscle to prevent delivering positive animals to slaughterhouses. LoD was established as the lowest level at which all samples gave a positive result. According to ISO 13969:2003, at least 3–5 replicates for each substance/level combination need to be performed. To set the LoD in the present study, at least 8 spiked samples at the level of interest were tested (Table S1).

Table 2 summarizes the LoD for swine blood serum obtained from those analyses with Explorer^®^-Blood test coupled to e-Reader^®^. In general, the e-Reader^®^ values obtained for the tested antimicrobials were far removed from the cut-off level (set to 55 units), which indicates that the system is thoroughly reliable in distinguishing positive from negative samples, and that the LoD for certain molecules could be set even lower. The corresponding LoD for muscle was also included for comparison. As MRLs have not been established for blood, those established by EU legislation for muscle were used as a reference. A satisfactory equivalence has been found between antibacterial concentration in muscle and blood serum for some antimicrobials (oxytetracycline and enrofloxacin), but not for others (sulfamethoxypyridazine), which correlated well but had higher concentrations in blood than in muscle [25]. Moreover, data from the validation of the Explorer^®^-Blood test showed a LoD for sulfonamides equivalent to the LoD obtained when applying Explorer^®^ to meat [15]. Even though a false positive could appear due to a higher concentration of sulfonamides in blood compared to muscle, this should not be regarded as a concern that would affect consumer safety.

### 3.4. Validation with Blood Samples Injected In Vivo

Beyond the LoDs established with spiked samples, the method’s performance was assessed with serum samples injected in vivo, containing residues of amoxicillin, oxytetracycline and sulfamethoxypyridazine. Samples with levels of amoxicillin below the LoD of the confirmatory method gave negative results with Explorer^®^-Blood (Table 3). Conversely, samples with higher levels of amoxicillin gave positive results. However, 2 samples with initial levels of 58 and 73 ppb as determined by LC-MS/MS were negative to Explorer^®^-Blood analysis. This result was unexpected, since the LoD for amoxicillin with Explorer^®^-Blood is much lower (10–15 ppb), and it is coherent with the LoDs found previously for muscle [15]. Therefore, these samples were re-analysed with LC-MS/MS in order to verify any degradation of antibiotics, as they had been kept frozen for 18 months before the analyses with Explorer^®^-Blood were performed. Confirmatory analysis demonstrated that amoxicillin residues had been degraded, as amoxicillin was no longer detected by LC-MS/MS. To our knowledge, no study of the stability of antibiotics in blood serum has been previously reported. However, the stability of antibiotics in meat can indeed be low in some cases [7,33,34] and could thus compromise confirmatory analysis results. Confirmation of presumptive positive results should therefore be carried out as soon as possible after screening, as certain storage conditions such as freezing temperature, time of storing and sample preparation may have an impact on antimicrobial stability [33].

Table 4 shows the results obtained for oxytetracycline: samples with high levels were adequately detected by Explorer^®^-Blood. Even some samples with oxytetracycline levels around the EU-MRL value for muscle could be detected (71 and 102 ppb), while others could not (83 and 89 ppb). These results are consistent with the LoD determined for Explorer^®^-Blood by spiking antibiotic-free sera with oxytetracycline, where only half of the replicates were positive at 100 ppb, while 100% of replicates gave positive results at 200 ppb.

In the case of sulfamethoxypyridazine, positive results were found even below 50 ppb (Table 5). This molecule was not included in the spiking study previously described; the result is nevertheless consistent with the LoD for other sulfonamides included in the validation study, where they were detected at 100 ppb (100% of replicates), thereby indicating that some positive replicates could be obtained at lower levels.

**Figure 5:**
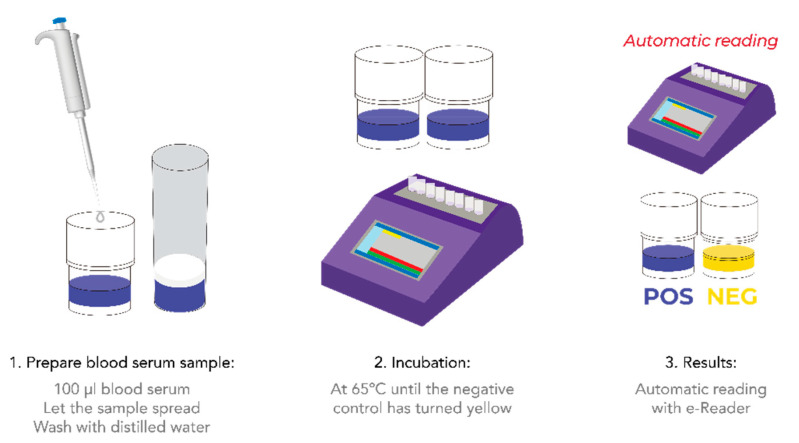
Schematic representation of the procedure for the detection of antibiotic residues in blood serum.

Overall, and thanks to the described procedure (Figure 5) and the validation carried out, Explorer^®^-Blood is proposed as a new, accurate tool for the in vivo detection of antibacterial residues, useful in preventing non-compliant animals from being delivered to slaughterhouses and thus avoiding their entrance into the food chain.

## 4. Conclusions

A post mortem method for the screening of antimicrobials in meat has been adapted as an ante mortem tool for the analysis of antibiotics and sulfonamides in blood serum. The new method was validated and compared with results obtained in meat, thereby proving that the Explorer^®^-Blood test coupled to e-Reader^®^ is a suitable method for ante mortem implementation at farm or slaughterhouse level. On the one hand, blood serum proved to be the ideal matrix, as it is easy to obtain and prepare for analysis and its antimicrobial concentrations showed a satisfactory equivalence with muscle in the case of most antimicrobials [25]. On the other hand, this method’s adaptation only required a small number of modifications, thereby maintaining its ease of analysis. The new Explorer^®^-Blood test can be carried out by non-qualified personnel, at any location, and within a brief time interval. Hence, it can be regarded as a pioneering tool for the analysis of antibacterial residues in living animals, thereby ensuring the absence of such residues in meat while preserving the agricultural production economy as well as consumer health.

## Figures and Tables

**Table 1:** Antimicrobial substances and levels selected to determine the LoDs of Explorer^®^ coupled to e-Reader^®^ for blood serum. The table additionally shows the EU-MRLs for muscle.

Molecule	Concentration Level Tested (µg/L)	MRL (Muscle)
Amoxicillin	10, 15, 20	50
Cefalexin	100, 200, 250, 300, 400	200
Ceftiofur	100, 200, 300, 400	1000
Sulfamethazine	100, 150, 200	100
Sulfadiazine	50, 100, 150	100
Oxitetracycline	100, 200, 300	100
Doxicyclin	100, 200, 300, 400	100
Neomycin	5, 15, 25, 50	500
Apramycin	500, 1000, 1500	1000
Tylosin	25, 50, 75	100
Lincomycin	200, 300, 400	100

**Table 2:** Limits of detection (LoD) of Explorer^®^-Blood test coupled to e-Reader^®^ obtained for several molecules from different families of antibacterial compounds in blood serum. LoD for muscle (Mata et al., 2014) and the corresponding MRL (Commission Regulation (EU) No. 37/2010)) are also included for comparison. e-Reader^®^ values are presented as the mean ± standard deviation (SD) of at least 8 independent replicates. LoD and MRL are presented as µg Kg^−1^.

Molecule	e-Reader^®^ Value	LoD Serum	LoD Muscle	MRL (Muscle)
Amoxicillin	121 ± 7	10	10	50
Cefalexin	67 ± 5	200–250	200	200
Ceftiofur	103 ± 9	300	200	1000
Sulfamethazine	76 ± 7	100	100	100
Sulfadiazine	89 ± 4	100	50	100
Oxitetracycline	80 ± 6	200	200	100
Doxicyclin	80 ± 7	100	100	100
Neomycin	99 ± 6	25	≤200	500
Apramycin	123 ± 4	≤500	900	1000
Tylosin	92 ± 3	25–50	100	100
Lincomycin	65 ± 3	200	300	100

**Table 3:** Explorer^®^-Blood test coupled to e-Reader^®^ values obtained for blood serum samples injected in vivo with amoxicillin and analysed by LC-MS/MS. The qualitative positive (+)/negative (−) result is also shown in the table. Cut-off = 55.

Sample	LC-MS/MS (µg Kg^−1^)	e-Reader^®^ Value	Qualitative Result
1	<10	45	−
2	<10	47	−
3	73/ND *	41	−
4	<10	45	−
5	<10	48	−
6	<10	37	−
7	58/ND *	36	−
8	<10	37	−
9	<10	39	−
10	262	129	+
11	2005	121	+
12	872	126	+
13	1484	121	+

* ND: non detected by LC-MS/MS at the time the samples were analysed with Explorer^®^ Blood.

**Table 4:** Explorer^®^-Blood test coupled to e-Reader^®^ values obtained for blood serum samples injected in vivo with oxytetracycline and analysed by LC-MS/MS. The qualitative positive (+)/negative (−) result is also shown in the table. Cut-off = 55.

Sample	LC-MS/MS (µg Kg^−1^)	e-Reader^®^ Value	Qualitative Result
1	403	94	+
2	<10	40	−
3	375	87	+
4	234	70	+
5	220	86	+
6	71	57	+
7	83	53	−
8	89	50	−
9	46	52	−
10	24	33	−
11	37	35	−
12	102	57	+
13	55	48	−
14	41	50	−
15	32	32	−
16	58	34	−

**Table 5:** Explorer^®^-Blood test coupled to e-Reader^®^ values obtained for blood serum samples injected in vivo with sulfamethoxypyridazine and analysed by LC-MS/MS. The qualitative positive (+)/negative (−) result is also shown in the table. Cut-off = 55.

Sample	LC-MS/MS (µg Kg^−1^)	e-Reader^®^ Value	Qualitative Result
1	1095	115	+
2	133	94	+
3	51	74	+
4	11	35	−
5	4098	117	+
6	773	112	+
7	569	114	+
8	115	89	+
9	25	102	+
10	3462	111	+
11	1113	109	+
12	229	101	+
13	74	82	+
14	35	65	+

## Data Availability

Data is contained within the article and Supplementary Materials.

## Supplementary Materials

The following are available online at https://www.mdpi.com/article/10.3390/foods10081897/s1, Table S1: e-Reader^®^ values obtained with Explorer^®^-Blood for the different combinations of antimicrobials and concentrations tested (spiked samples). Each column (R1-R10) presents the result of each of the replicates analyzed, as well as de medium value, standard deviation (SD) and qualitative result (+/−). For comparison purposes and as a reference, the last column includes the EU-MRLs for muscle (Commission Regulation (EU) No. 37/2010). Concentration is presented as µg Kg^−1^. Concentrations in bold correspond to the LoDs of the method.
