# Supplementary material for: Optimization and Validation of a New Microbial Inhibition Test for the Detection of Antimicrobial Residues in Living Animals Intended for Human Consumption
Source: Foods. 2021 Aug 16;10(8):1897. doi: 10.3390/foods10081897 (PMC8393348; doi:10.3390/foods10081897)
Supplement: Supplementary file 1 [file foods-10-01897-s001.zip › foods-1265765-supplementary.pdf]

foods

MDPI

Table S1. e-Reader® values obtained with Explorer®-Blood for the different combinations of antimicrobials and concentrations tested (spiked samples). Each column (R1-R10) presents the result of each of the replicates analyzed, as well as de medium value, standard deviation (SD) and qualitative result (+/−). For comparison purposes and as a reference, the last column includes the EU-MRLs for muscle (Commission Regulation (EU) No 37/2010). Concentration is presented as μg Kg^{-1}. Concentrations in bold correspond to the LoDs of the method.

Molecule	Concentration	n	R1	R2	R3	R4	R5	R6	R7	R8	R9	R10	e-Reader® Value	SD	Result	EU-MRL (Muscle)
	10	3	76	82	80								79	3	+	
Amoxicillin	**15**	8	117	117	117	109	125	127	129	129			121	7	**+**	**50**
	20	3	127	123	124								125	2	+	
	100	5	34	39	38	38	35						37	2	−	
	200	10	73	74	71	50	43	49	66	64	107	105	70	22	+/−	
Cefalexin	**250**	8	67	73	76	65	65	65	60	65			67	5	**+**	**200**
	300	5	75	76	73	116	112						90	22	+	
	100	3	31	32	32								32	1	−	
	200	4	33	33	35	62							41	14	−	
Ceftiofur	**300**	8	107	118	102	110	104	99	92	90			103	9	**+**	**1000**
	400	3	121	117	120								119	2	+	
	**100**	8	65	65	79	81	81	83	75	76			76	7	**+**	**100**
Sulfamethazine	150	7	82	80	91	92	80	79	94				85	7	+	
	200	8	83	78	90	90	87	88	86	93			87	5	+	
	50	3	55	50	54								53	3	−	
Sulfadiazine	**100**	8	94	89	89	88	87	82	90	94			89	4	**+**	**100**
	150	3	95	99	97								97	2	+	
	100	8	60	57	51	54	57	56	52	51			55	3	+/−	
Oxytetracycline	**200**	8	84	77	82	80	91	74	78	74			80	6	**+**	**100**
	300	3	98	96	98								97	1	+	
	**100**	8	77	78	70	77	84	78	87	91			80	7	**+**	**100**
	200	8	60	65	102	95	98	102	102	96			90	17	**+**	

Doxycycline	300	8	91	97	114	111	113	111	114	114			108	9	+	
	400	3	112	108	110								110	2	+	
Neomycin	15	3	70	79	76								75	5	+	
	**25**	10	103	106	97	87	101	99	96	105	95	96	99	6	**+**	**500**
	50	3	110	115	114								113	3	+	
Apramycin	**500**	8	122	120	117	127	127	122	125	122			123	3	**+**	**1000**
	1000	3	126	130	129								128	2	+	
	1500	3	128	125	128								127	2	+	
Tylosin	25	3	59	58	60								59	1	+	
	**50**	8	94	92	95	91	95	93	91	87			92	3	**+**	**100**
	75	3	110	97	104								104	7	+	
Lincomycin	**200**	8	65	66	67	62	66	69	58	65			65	3	**+**	**100**
	300	8	78	77	71	66	82	71	72	70			73	5	+	
	400	3	78	79	78								78	1	+	
